# Novel Monoclonal Antibodies Against Mouse C1q: Characterisation and Development of a Quantitative ELISA for Mouse C1q

**DOI:** 10.1007/s12035-021-02419-5

**Published:** 2021-05-18

**Authors:** Robert A. J. Byrne, Megan Torvell, Nikoleta Daskoulidou, Dina Fathalla, Eirini Kokkali, Sarah M. Carpanini, B. Paul Morgan

**Affiliations:** 1 UK Dementia Research Institute Cardiff, Hadyn Ellis Building, Cardiff University, Maindy Road, Cardiff, CF244HQ UK; 2 Division of Infection and Immunity and Systems Immunity Research Institute, School of Medicine, Cardiff University, Hadyn Ellis Building, Heath Park, Cardiff, CF144XN UK; 3 School of Optometry and Visual Sciences, Cardiff University, Maindy Road, Cardiff, CF244HQ UK

**Keywords:** Complement, C1q, ELISA, Mouse, Alzheimer’s

## Abstract

Recent studies have identified roles for complement in synaptic pruning, both physiological during development and pathological in Alzheimer’s disease (AD). These reports suggest that C1q initiates complement activation on synapses and C3 fragments then tag them for removal by microglia. There is an urgent need to characterise these processes in rodent AD models; this requires the development of reagents and methods for detection and quantification of rodent C1q in fluids and pathological tissues. These will enable better evaluation of the role of C1q in disease and its value as disease biomarker. We describe the generation in C1q-deficient mice of novel monoclonal antibodies against mouse and rat C1q that enabled development of a sensitive, specific, and quantitative ELISA for mouse and rat C1q capable of measuring C1q in biological fluids and tissue extracts. Serum C1q levels were measured in wild-type (WT), *C1q* knockout (KO), *C3* KO, *C7* KO, *Crry* KO, and 3xTg and APP^NL-G-F^ AD model mice through ageing. C1q levels significantly decreased in WT, APP^NL-G-F^, and C7 KO mice with ageing. C1q levels were reduced in APP^NL-G-F^ compared to WT at all ages and in 3xTg at 12 months; *C3* KO and *C7* KO, but not *Crry* KO mice, also demonstrated significantly lower C1q levels compared to matched WT. In brain homogenates, C1q levels increased with age in both WT and APP^NL-G-F^ mice. This robust and adaptable assay for quantification of mouse and rat C1q provides a vital tool for investigating the expression of C1q in rodent models of AD and other complement-driven pathologies.

**Supplementary Information:**

The online version contains supplementary material available at 10.1007/s12035-021-02419-5.

## Introduction

Alzheimer’s disease (AD) is a severe neurodegenerative disorder characterised by amyloid-ß (Aβ) plaques, neurofibrillary tangles of hyperphosphorylated tau, neuroinflammation, synaptic loss, and cognitive decline. Multiple lines of evidence, genetic and experimental, have implicated the complement system in the aetiology of AD. Genome-wide association studies (GWAS) have repeatedly associated single nucleotide polymorphisms (SNPs) in genes encoding complement regulators clusterin (*CLU*) and complement receptor 1 (*CR1*) with risk of late-onset AD [1–4]. Biomarker studies have identified altered levels of complement proteins and activation products in plasma and/or cerebrospinal fluid (CSF) that distinguish AD patients from controls and predict progression from mild cognitive impairment (MCI) to AD [5–9]. Microarray studies identified upregulation of numerous complement genes in AD patients versus age-matched controls [10]. Immunohistochemical analysis of post-mortem AD brains identified deposition of complement proteins and activation products, including C1q, C3, and C4, in and around plaques and tangles [11–13]. Furthermore, deletion of C3 or suppression of C3 activation in multiple AD mouse models increased amyloid burden, indicating that complement is involved in amyloid clearance [14–16].

The complement system is the innate immune system’s primary defence against invading pathogens. In excess of 30 soluble and membrane-bound proteins, three distinct pathways coordinate to promote inflammation and destruction of pathogens. The classical pathway of complement activation is initiated when the circulating C1 complex binds to target surfaces via its C1q subunit, a heterohexameric defence collagen that recognises immunoglobulin Fc domains [17]. The other C1 components C1r and C1s associate with the collagen stalks of C1q, the latter catalysing proteolysis of C4 and C2 to form the classical pathway C3 convertase [18]; this then cleaves the central complement component C3 into C3a and C3b with multiple downstream consequences including opsonisation for phagocytosis, generation of the inflammatory mediator C5a, and formation of the membrane attack complex (MAC) [19–21].

Previous studies have highlighted a critical role for the classical complement cascade in physiological synaptic pruning during development. C1q and C3 fragments (C3b/iC3b) localise to and tag specific synapses for removal in the developing rodent visual system and substantial synaptic pruning defects were demonstrated in *C1q* knockout (KO), *C3* KO, and *C4* KO mice [22–24]. It is established that microglia are responsible for phagocytosis of synapses during developmental pruning, a process that involves interaction of complement receptor 3 (CR3; CD11b/CD18) expressed on the surface of microglia and its ligand iC3b on target synapses [25, 26].

Synaptic elimination also occurs pathologically, and is an early event in the pathogenesis of AD, occurring up to 20 years prior to the onset of cognitive dysfunction [27]. Studies in AD rodent models have implicated the classical pathway in pathological synaptic loss; C1q is deposited on synapses destined for elimination, and synaptic elimination is reduced or abolished by either *C1q* deletion or blocking of C1q with inhibitory antibodies in both amyloid and tau models [28, 29]. Microglia from *CR3* KO mice displayed impaired synaptic engulfment triggered by local administration of oligomeric Aß [28]. Pathological synaptic pruning driven by complement is also reported in multiple sclerosis (MS) [30, 31] and in schizophrenia, where dysregulated *C4* expression contributes to abnormal synaptic pruning [24, 32, 33].

While the precise mechanism of synaptic loss in AD remains conjecture, the prevailing hypothesis is that the developmental synaptic pruning process is reactivated and becomes dysregulated, resulting in inappropriate classical pathway activation and microglial phagocytosis of complement-opsonised synapses; hence, there is a pressing requirement for reliable and reproducible methods for measuring expression of C1q and other classical pathway components in fluids and pathological tissues in models and man. We have generated a panel of monoclonal antibodies against rodent C1q and developed an enzyme-linked immunosorbent assay (ELISA) that allows specific and quantitative measurement of mouse and rat C1q protein levels in serum and in brain extracts. We demonstrate that the ELISA identifies age-related changes in C1q concentration and differences in C1q levels between wild-type (WT) mice, complement KO mice and the APP^NL-G-F^ and 3xTg mouse models of AD.

## Materials and Methods

Reagents and chemicals were purchased from Thermo Fisher Scientific (Paisley, UK) unless stated otherwise. Composition of phosphate-buffered saline (PBS) is 137 mM NaCl, 2.7 mM KCl, 10 mM Na_2_HPO_4_, 1.8 mM KH_2_PO_4_, pH 7.4. All dialysis was performed overnight at 4 °C with 12–14-kDa cut-off dialysis tubing (Medicell, London, UK). All cells were cultured at 37 °C in 5% CO_2_. All protein stain and Western blot images were captured using the G:BOX Chemi XX6 (Syngene, Cambridge, UK).

### Animals

All procedures complied with UK Animals Scientific Procedures Act 1986 and local regulations. All animals were group-housed in environmentally enriched cages, under standard pathogen-free conditions, with a 12-h light/dark cycle, and access to food and water ad libitum. C57BL/6 (WT; Harlan, Bicester, UK), *C1q* KO [34], *C3* KO [35], *C7* KO (Jackson ImmunoResearch), *Crry* KO [36], APP^NL-G-F^ [37], and 3xTg [38] mice have been described elsewhere. With the exception of the 3xTg line which is on a mixed background, all of the mouse lines were on the C57BL/6 background and all had been back-crossed onto in-house WT mice; all were maintained in the same room in the facility at the same level of containment.

All mice were humanely euthanised by increasing CO_2_ concentration, death was confirmed by palpation, and whole blood was collected by transcardial puncture. WT and APP^NL-G-F^ mice were perfused with cold PBS; brains were removed, and snap-frozen. Blood samples were taken from 3-month-old male Brown Norway rats (Envigo, Bicester, UK) by tail tipping under anaesthesia. Collected mouse and rat blood was allowed to clot at room temperature for 10 min, placed on ice for 1 h, then centrifuged; serum was removed and immediately stored in aliquots at −80 °C.

Mouse brain homogenates were obtained using standard methods. In brief, frozen mouse brains (one hemisphere) were homogenised in a Dounce glass homogeniser in 1 ml of 5 mM KCl, 1 mM MgCl_2_, 25 mM HEPES, 120 mM NaCl, and 2 mM CaCl_2_ (pH 7.5), supplemented with complete mini EDTA-free protease inhibitors (Roche, Welwyn Garden City, UK), and phosphatase inhibitor cocktail V (Millipore, Watford, UK). Homogenates were passed through 80-μm nylon filters (Millipore). Samples were lysed on ice in RIPA buffer (Sigma-Aldrich, Gillingham, UK) containing protease inhibitors. The resultant lysate was centrifuged at 17,000×*g* for 10 min at 4 °C and supernatant collected. Total protein concentration was measured using the Pierce BCA protein assay kit.

### Isolation of Mouse C1q from Mouse Serum

C1q was isolated from mouse serum via a three-step protocol on an ÄKTA pure chromatography system (GE Healthcare, Amersham, UK). Elements of the protocol were derived from published methods [39, 40]. Sterile mouse serum (TCS Biosciences, Claydon, UK) was 0.22-μm-filtered and diluted 1:1 in binding buffer (20 mM Tris, 120 mM NaCl, 20 mM EDTA, pH 7.0) to dissociate C1 complexes. Human IgG (10 mg) was immobilised on a 5-ml HiTrap NHS-Activated HP column (GE Healthcare; manufacturer’s protocol); rabbit anti-human IgG antiserum was passed over this column to saturate binding sites. The column was washed and equilibrated with 5 column volumes (CV) of binding buffer. Filtered mouse serum was applied to the column, the column was washed, and bound C1q was eluted with 3 CV of elution buffer (50 mM Tris, 1 M NaCl, 20 mM EDTA, pH 10). In order to remove contaminating mouse immunoglobulins, the eluate was applied to a 5-ml HiTrap Protein G HP column (GE Healthcare; manufacturer’s instructions), the flow-through collected, dialysed into cation exchange binding buffer (20 mM HEPES, 60 mM NaCl, 10 mM EDTA, pH 7.8), and applied to a 5-ml HiTrap SP HP column (GE Healthcare) equilibrated with 5 CV of cation exchange binding buffer. Proteins were eluted with an increasing salt gradient in cation exchange elution buffer (to 700 mM NaCl) over 20 CV. Fractions containing eluted protein were dialysed into 50 mM NaH_2_PO_4_ and 100 mM NaCl (pH 7.4), and concentrated to 0.5 ml using a 30-kDa Vivaspin 6 centrifugal concentrator (Sartorius, Epsom, UK); concentration was measured using Bradford reagent according to the manufacturer’s protocol (Sigma-Aldrich) and pure C1q-containing fractions identified by SDS-PAGE on in-house 12.5% tris-glycine gels in the presence and absence of *β*-mercaptoethanol and stained with Coomassie (0.25% (*w*/*v*) Coomassie Brilliant Blue R-250, 40% (*v*/v) methanol, 10% (v/v) acetic acid).

The novel anti-C1q monoclonal antibodies (mAb) were also used to affinity-purify mouse C1q in a single step. The mAb (3 mg) was conjugated to a 1-ml HiTrap NHS-activated HP column (GE Healthcare; manufacturer’s protocol), pooled mouse serum was 0.22-μm-filtered and diluted 1:1 in binding buffer (10 mM Tris, 150 mM NaCl, 20 mM EDTA, pH 7.4). The mAb column was equilibrated with 5 CV of binding buffer. Filtered mouse serum was applied to the column, the column was washed in binding buffer, and bound C1q was eluted with 3 CV of elution buffer (100 mM glycine, 20 mM EDTA, pH 3). C1q-containing fractions were identified, dialysed, concentrated, quantified, and examined via SDS-PAGE as described above.

### Immunisation, Generation, and Isolation of Hybridoma

*C1q* KO mice (8–16 weeks of age) were immunised subcutaneously with 50-μg mouse C1q in complete Freund’s adjuvant, boosted after 4 weeks with 50-μg mouse C1q in incomplete Freund’s adjuvant and then repeat boosted 1 week later. A week after the second boost, mice were tail-bled, serum-harvested, and screened for immunoreactivity against mouse C1q via direct ELISA as described below. Mice with the strongest immune response then received an intra-peritoneal boost of 50 μg C1q in PBS and were humanely sacrificed 2 days later, and spleens were removed. Splenocytes were extracted and fused with SP2/0-Ag14 mouse myeloma cells (European Collection of Animal Cell Cultures, Salisbury, UK) using an established protocol [41].

Cells from the fusion were grown in RPMI 1640 medium containing 2% penicillin/streptomycin, 2 mM glutamine, 1 mM sodium pyruvate, and 15% foetal bovine serum (FBS), supplemented with HAT (Gibco, Paisley, UK) to select hybridomas, in 96-well plates (10 plates per fusion) with *C1q* KO mouse peritoneal macrophages as feeder cells. After 14 days, medium was harvested from each well and screened for C1q immunoreactivity via direct ELISA as described below. Positive clones were sub-cloned three times by limiting dilution to ensure monoclonality, and ELISA was used for screening at each cloning to verify C1q specificity. Positive monoclonal hybridomas were then isotyped using an IsoStrip™ Mouse Monoclonal Antibody Isotyping Kit (Roche) and cultured in CELLine™ 1000 bioreactor flasks (VWR, Lutterworth, UK) with RPMI 1640 medium containing 2% penicillin/streptomycin, 2 mM glutamine, 1 mM sodium pyruvate, and 10% ultra-low IgG FBS (Gibco). Bioreactor flasks were harvested every 7–10 days. IgG mAb were purified using a 5-ml HiTrap Protein G HP column (GE Healthcare; manufacturer’s instructions); IgM mAb were purified using a 1-ml HiTrap Protein L column (GE Healthcare; manufacturer’s instructions). All mAb were dialysed into PBS. Selected mAb were conjugated to biotin using the EZ-Link™ Sulfo-NHS-LC-Biotin reagent (Sigma-Aldrich; manufacturer’s protocols).

### Screening for Antibodies Specific to Mouse C1q

Pure mouse C1q was diluted to 0.5 μg/ml in carbonate buffer (100 mM NaHCO_3_, 100 mM Na_2_CO_3_, pH 9.6), dispensed (50 μl/well) into Nunc MaxiSorp™ flat-bottom 96-well plates (Invitrogen, Paisley, UK), and incubated overnight at 4 °C. Wells were blocked with 2% (*w*/*v*) bovine serum albumin (BSA) in PBS-T (100 μl/well) for 1 h at 37 °C, washed twice with PBS-T, and either serum from immunised mice (diluted 1:100 in 0.2% BSA-PBS-T) or neat hybridoma supernatant (50 μl/well) added and incubated for 90 min at 37 °C. Wells were washed again with PBS-T and then incubated with a 1:1000 dilution (50 μl/well) of peroxidase-conjugated goat anti-mouse IgG (H + L) secondary antibody (Jackson ImmunoResearch) for 30 min at 37 °C. Following two final washes, plates were developed using O-phenylenediamine dihydrochloride (SIGMAFAST™ OPD, Sigma-Aldrich). Absorbance was measured at 492 nm with a FLUOstar™ Omega Microplate Reader (BMG LABTECH).

### ELISA Characterisation of Novel mAb

Anti-C1q mAb were diluted to 5 μg/ml in carbonate buffer and coated onto Nunc MaxiSorp™ flat-bottom 96-well plates (50 μl/well) for 1 h at 37 °C. Blocking and washing was performed as described above. Mouse (WT and *C1q* KO), rat, and human sera were loaded in a dilution series from 1 to 0.001% and incubated at 37 °C for 2 h. Bound C1q was detected using rabbit anti-human C1q polyclonal antibody (2 μg/ml; in-house) and peroxidase-conjugated donkey anti-rabbit IgG secondary antibody (1:5000; Jackson ImmunoResearch, West Grove, PA, USA). OPD development was performed as detailed above.

### Western Blotting

Pure mouse C1q (1 μg) was run on in-house 12.5% tris-glycine gels in the presence and absence of *β*-mercaptoethanol, then transferred onto nitrocellulose 0.45-μm membrane (GE Healthcare) using a Mini Blot Module (Invitrogen). Membranes were blocked for 1 h at room temperature with 5% (*w*/*v*) BSA in PBS-T, incubated with mouse anti-C1q antiserum (1:500) or anti-C1q mAb (2 μg/ml) overnight at 4 °C and developed using peroxidase-conjugated donkey anti-mouse IgG (H + L) secondary antibody (1:5000; Jackson ImmunoResearch) followed by ECL Western blotting detection reagent (GE Healthcare).

To validate the ELISA, pure mouse C1q (200 ng) and sera (diluted 1:10 in PBS, 10 μl loaded) from C1q KO, WT, and APP^NL-G-F^ mice (3 and 12 months) were analysed under reducing conditions as described above. Membranes were stained with Ponceau S immediately after transfer according to the manufacturer’s instructions (Sigma-Aldrich). Following blocking, membranes were incubated with rabbit anti-C1q mAb (2 μg/ml; Abcam, Cambridge, UK) overnight at 4 °C and developed using peroxidase-conjugated donkey anti-rabbit IgG (H + L) secondary antibody (1:5000; Jackson ImmunoResearch). Densitometry was performed using GeneTools (Syngene).

### Haemolytic Assays

Sheep erythrocytes were isolated from blood (TCS Biosciences) and antibody sensitized as described [42]. For assays involving mouse serum, sensitised sheep erythrocytes were additionally sensitised with 20 μg/ml mouse anti-rabbit IgG (Invitrogen). Characterisation of novel mAb in human, rat, and mouse haemolytic assays was performed according to published methods [43]. To assess the function of immunoaffinity-purified mouse C1q, a titration of purified protein (25 > 0 μg/ml) was added to 25% *C1q* KO mouse serum and then tested in haemolysis assays as above.

### Development of a Sandwich ELISA for Quantification of Mouse C1q

The anti-C1q mAb selected for capture was diluted to 5 μg/ml in carbonate buffer and coated onto Nunc MaxiSorp™ flat-bottom 96-well plates (50 μl/well) for 1 h at 37 °C. Blocking and washing were performed as described above. Pure C1q standards in a dilution series from 2 μg/ml > 2 ng/ml were loaded in duplicate to generate a standard curve. To measure levels in serum (mouse and rat) and mouse brain homogenate, samples were analysed in triplicate (50 μl/well) at dilutions of 1:800 for serum and 0.5 mg/ml total protein for brain homogenates, respectively. Inter-assay controls were included on all plates. All samples and standards were incubated for 2 h at 37 °C then washed twice with PBS-T. The anti-C1q mAb selected for detection (biotinylated) was diluted in 0.2% (*w*/*v*) BSA-PBS-T (2 μg/ml, 50 μl/well), added, and incubated for 1 h at 37 °C. After washing, wells were incubated (1 h, 37 °C) with streptavidin-HRP (1:200; R&D Systems, Abingdon, UK) diluted in 0.2% (w/v) BSA-PBS-T (50 μl/well). After two final washes, colour was developed using OPD and absorbance measured as above. C1q concentrations in serum and brain samples were interpolated from the standard curve using Prism 5 (GraphPad, La Jolla, CA, USA).

To test assay performance, ten WT mouse serum samples (age 3 months, male *n* = 6, female *n* = 4) were analysed using the above protocol multiple times on different days and either with or without addition of 10 mM EDTA to dissociate C1. To test whether the assay could detect C1q in CSF, human CSF (hCSF) was supplemented with a known quantity of pure mouse C1q and then measured using the assay. Spike recovery was performed by adding a known amount of pure mouse C1q to WT mouse serum prior to dilution and then measuring levels in the base and supplemented serum using the assay as above. Spike recovery was calculated as a percentage of calculated recovery relative to expected recovery. Inter- and intra-assay coefficients of variability (%CV) were derived from the multiple measurements by standard methods [44].

### Statistical Analyses

All statistical analyses were performed using GraphPad Prism 5. Data points that fell 1.5× the interquartile range above or below the mean were considered outliers and excluded. All data was checked for normality with the Kolmogorov-Smirnov test. The paired *t-*test was used to test for differences in the same serum samples in the presence or absence of EDTA. The unpaired *t*-test was used to test for differences in serum C1q levels between age groups of the same complement KO genotype, and for differences in brain C1q levels between age groups of the WT and APP^NL-G-F^ genotypes. One-way analysis of variance (ANOVA) was utilised to test for differences between age groups of the WT and APP^NL-G-F^ genotypes, two-way ANOVA was used to test for differences between genotypes at each age. Tukey’s post hoc test and the Bonferroni post hoc test were co-implemented with one-way ANOVA and two-way ANOVA, respectively. *P* < 0.05 was considered significant. C1q levels are described in the results as mean ± standard deviation.

## Results

### Purification of Mouse C1q and Generation of mAb

**Fig. 1:**
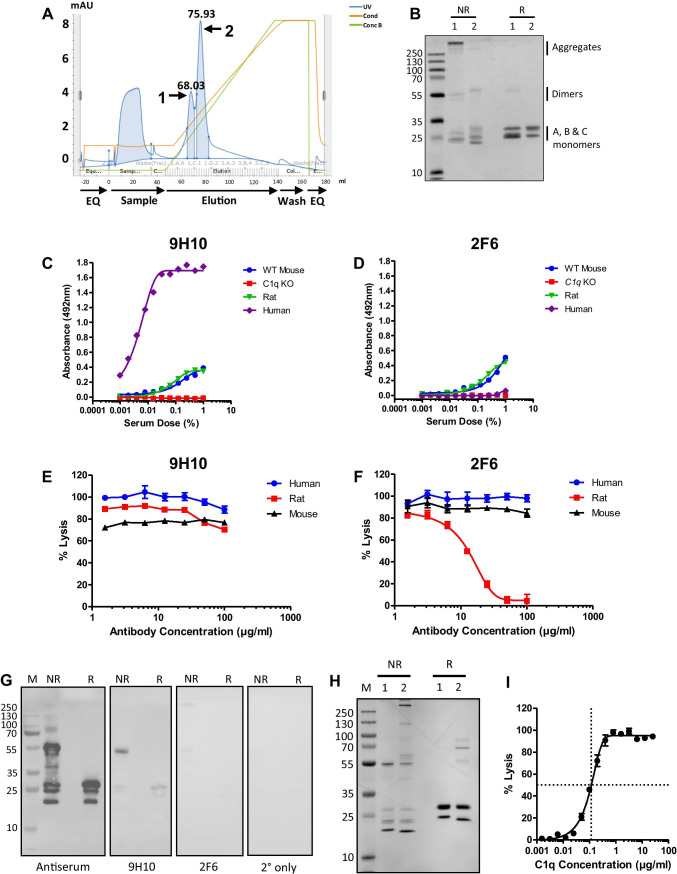
Isolation of pure mouse C1q and characterisation of novel monoclonal antibodies. **(A)** Representative ÄKTA chromatogram from the cation exchange purification of mouse C1q showing 2 peaks. **(B)** SDS-PAGE of cation exchange fractions (2.5 μg/lane) from peak 1 and peak 2; non-reduced (NR), or reduced with 5% *β*-mercaptoethanol (R), proteins were stained with Coomassie blue. Peak 1 contained aggregated mouse C1q that reduced to the individual C1q A, B, and C monomers at 31 kDa, 29 kDa, and 26 kDa; peak 2 contained pure mouse C1q with no aggregates that reduced to C1q monomers. **(C)** C1q sandwich ELISA using 9H10 as capture antibody and polyclonal anti-C1q as detect showing cross-reactivity with mouse, rat, and human C1q. **(D)** C1q sandwich ELISA using 2F6 as capture antibody and polyclonal anti-C1q as detect showing 2F6 cross-reactivity with mouse and rat C1q but not human C1q. **(E**, **F)** Classical pathway haemolytic assays showing that 9H10 had no inhibitory activity towards mouse, rat, or human C1q, while 2F6 inhibited rat C1q, but not mouse or human C1q. **(G)** Western blot of mouse C1q. Denatured pure C1q (1 μg/lane) under NR and R conditions and Western blotted with mouse anti-C1q antiserum (1:500), 9H10, and 2F6 (2 μg/ml). 9H10 bound the A chain in denatured C1q under both non-reducing and reducing conditions; staining with 2F6 was weak under both conditions. All membranes were exposed together, and for the same time, the images are unmodified from the original .tif files. **(H)** SDS-PAGE of mouse C1q isolated via IgG affinity followed by cation exchange (1) and 9H10 immunoaffinity purification (2) under both non-reducing and reducing conditions and stained with Coomassie blue. **(I)** Classical pathway haemolytic assay demonstrating that titration (from 25 μg/ml) of mouse C1q isolated via 9H10 immunoaffinity restored activity to *C1q* KO serum (25%); 116 ng/ml of mouse C1q was required to restore lysis to 50%

C1q was purified from mouse serum using IgG affinity chromatography followed by cation exchange (Fig. 1A); C1q eluted from cation exchange as a double peak, the first peak containing predominantly aggregated C1q (Fig. 1A, B). The second peak was aggregate-free and pure on Coomassie-stained SDS-PAGE (Fig. 1B). Two fusions were performed; from 2880 wells screened, six hybridoma clones producing mouse C1q-specific antibodies were identified, five of which were IgM mAb; two of the clones, 9H10 and 2F6, gave consistently higher ELISA signal during screening and were taken forward for full characterisation (Table 1). Species cross-reactivity of 9H10 mAb (IgG2b isotype) and 2F6 mAb (IgM isotype) was evaluated via sandwich ELISA using a polyclonal anti-human C1q for detection. 9H10 cross-reacted with mouse, rat, and human C1q (Fig. 1C); 2F6 was specific for mouse and rat C1q only and gave no signal with human C1q (Fig. 1D). C1 inhibitory activity of 9H10 and 2F6 was assessed in haemolysis assays; 9H10 did not inhibit in any of the sera tested (Fig. 1E), while 2F6 only inhibited haemolysis in rat serum (Fig. 1F). In order to determine the C1q chain specificity of the mAbs, Western blotting was performed on purified mouse C1q. 9H10 predominantly recognised the A chain of C1q (~ 27 kDa) under reducing and non-reducing conditions; 2F6 was very weak in westerns, suggesting that its epitope was conformation-dependent (Fig. 1G). Immunoaffinity chromatography on 9H10 isolated C1q from mouse serum in a single step (Fig. 1H); the purified C1q restored activity to *C1q* KO mouse serum, demonstrating that it was fully functional (Fig. 1I).

**Table 1:** Novel C1q antibodies. Six antibodies were identified via direct ELISA screening against mouse C1q. 2F6 and 9H10 consistently gave higher signals in ELISA and were taken forward for further characterisation. The remaining antibodies were discontinued and hence not tested (N/T) for cross-reactivity

Antibody	Isotype	Cross-reactivity		
		Mouse	Rat	Human
1G1	IgM	+	N/T	N/T
2F6	IgM	+++	+++	–
7F3	IgM	++	N/T	N/T
7H2	IgM	+	N/T	N/T
9H10	IgG2b	+++	+++	+++
10G11	IgM	+	N/T	N/T

**Fig. 2:**
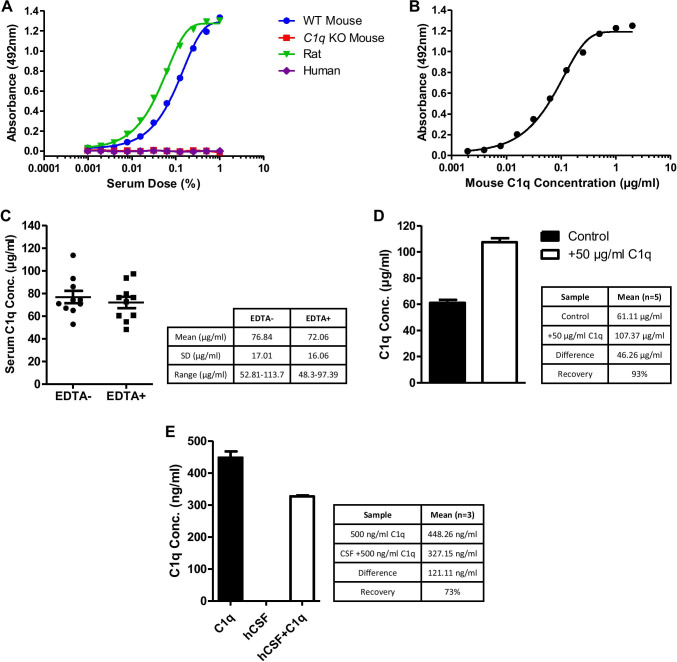
Optimisation and quality testing of in-house quantitative sandwich ELISA. **(A)** C1q sandwich ELISA using 9H10 mAb as capture and biotinylated 2F6 mAb as detection. Standard curves were generated by titrating WT mouse, *C1q* KO mouse, rat, and human serum from 1% serum concentration. The assay detected C1q in mouse and rat but not human serum. **(B)** Pure C1q was used as standard in the assay; detection range was 10–500 ng/ml. **(C)** Measurement of the same WT mouse serum samples (3 months, male *n* = 6, female *n* = 4) in the presence or absence of 10 mM EDTA to dissociate the C1 complex. EDTA had no significant effect on measured C1q levels. **(D)** Spike recovery. Mouse C1q (50 μg/ml) was added into WT mouse serum samples (3 months, male, *n* = 5) and C1q levels measured. Average recovery was 93%. **(E)** C1q spiked into human CSF (hCSF). Mouse C1q (500 ng/ml) was spiked into hCSF samples (*n* = 3; hCSF/C1q) or the same volume of buffer (C1q); C1q levels were measured. Unspiked hCSF was used as a control. Average recovery was 73%

A quantitative sandwich ELISA was developed using 9H10 mAb as capture and biotinylated 2F6 mAb as detection. The sandwich ELISA detected C1q in both mouse and rat sera but C1q-deficient mouse serum gave no signal in the assay (Fig. 2A). The assay did not detect C1q in human serum, expected as the 2F6 mAb was not reactive against human C1q; substitution of 2F6 with a polyclonal anti-C1q antibody enabled quantification of human C1q (not shown). Using pure mouse C1q as standard, the assay had a detection limit of 2 ng/ml and a working range of 15 ng/ml to 250 ng/ml (Fig. 2B). Dissociation of the C1 complex with EDTA had no significant effect on detection of C1q (Fig. 2C). Spike recovery was 93% (Fig. 2D), which was within acceptable parameters [45]. As expected, human CSF gave no signal in the assay; however, pure mouse C1q spiked into human CSF at levels similar to those reported for C1q in human CSF (200–500 ng/ml) [46] was readily detected with recovery of 73% (Fig. 2E). Two mouse serum samples, one with high and one with low C1q levels, were measured in triplicate across 10 assay plates to calculate an inter-assay coefficient of variability (%CV): for the “high” sample, mean of means = 56.40 μg/ml, SD = 4.19 μg/ml, CV = 7.44%; for the “low” sample, mean of means = 34.71 μg/ml, SD = 4.67 μg/ml, CV = 13.36%. The overall inter-assay CV was 10.45%. Sample replicates for all cohorts were used to generate an intra-assay %CV as described [47]; the intra-assay CV was 6.14%.

### Quantification of Serum C1q in WT Mice, AD Mice, and Complement KO Mice and Rats

**Fig. 3:**
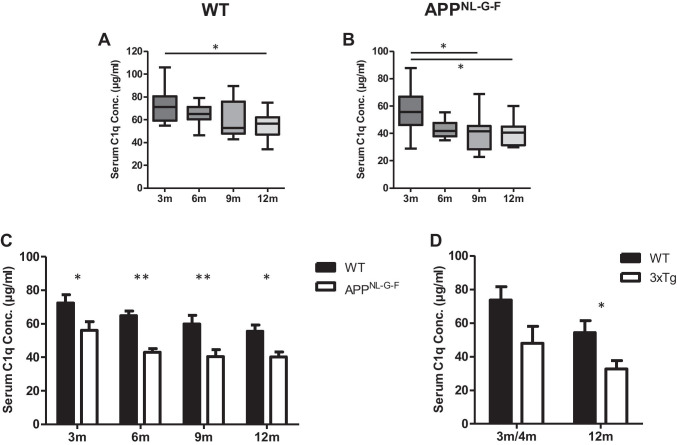
Measurement of C1q levels in WT and APP^NL-G-F^ mouse serum by quantitative sandwich ELISA. **(A)** Serum C1q levels measured in WT mice at 3, 6, 9, and 12 months. C1q levels were significantly decreased at 12 months compared to 3 months (*P* < 0.05). **(B)** Serum C1q levels measured in APP^NL-G-F^ mice at 3, 6, 9, and 12 months. Serum C1q levels were significantly reduced at 9 and 12 months compared to 3 months (*P* < 0.05). **(C)** Serum C1q levels were significantly lower in APP^NL-G-F^ mice than WT mice at all ages examined (* = *P* < 0.05, ** = *P* < 0.01). **(D)** C1q was measured in serum from male 3xTg and WT mice at 3-4 months (3xTg, *n* = 4; WT, *n* = 6) and at 12 months (3xTg, *n* = 5; T, *n* = 5). C1q levels were lower in 3xTg mice compared to WT at each age, significantly at 12 months (3-4 months, *p* = 0.078; 12 months, *p* = 0.038; *t* test)

**Fig. 4:**
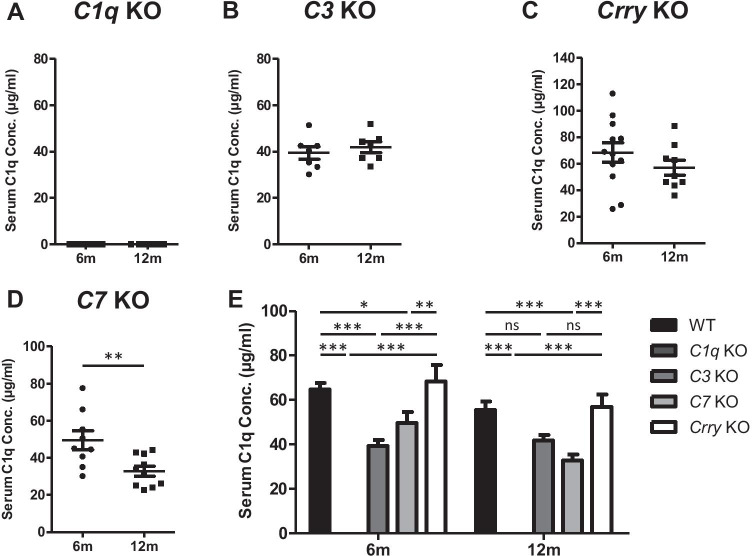
Measurement of C1q levels in *C1q* KO, *C3* KO, *Crry* KO, and *C7* KO mouse serum by quantitative sandwich ELISA. **(A)** Serum C1q levels were measured in *C1q* KO mice; no C1q was detected at 6 or 12 months. **(B**-**C)** Measurement of serum C1q levels in **B** *C3* KO and **C** *Crry* KO mice at 6 and 12 months of age; no significant differences in serum C1q levels were observed between 6 and 12 months (*P* > 0.05). **(D)** Measurement of serum C1q levels in *C7* KO mice; a significant decrease was identified at 12 months compared to 6 months of age (*P* < 0.01). **(E)** Serum C1q levels in *C3* KO, *C7* KO, and *C1q* KO were significantly reduced relative to WT and *Crry* KO at 6 months. At 12 months, serum C1q was only significantly reduced in *C7* KO and *C1q* KO mice compared to WT and *Crry* KO mice. There were no significant differences between WT and *Crry* KO mice. * = *P* < 0.05, ** = *P* < 0.01, *** = *P* < 0.001

The concentration of C1q in serum samples from WT and APP^NL-G-F^ mice aged 3, 6, 9, and 12 months was quantified using the sandwich ELISA (Table 2). In WT mice, serum C1q levels progressively decreased with age and were significantly decreased at 12 months compared to 3 months (Fig. 3A). APP^NL-G-F^ mice showed a more pronounced decrease with age, significant at both 9 and 12 months relative to 3 months (Fig. 3B). These observations were replicated via Western blotting with a commercial mAb (Figs. S1A, B). Serum C1q levels were significantly decreased in APP^NL-G-F^ mice compared to WT mice in all age groups (Fig. 3C); similar trends were observed at 4 and 12 months in the 3xTg AD mice compared to WT, reaching significance at 12 months (Fig. 3D). C1q levels in rat serum measured in the ELISA were 97.69 ± 14.31 μg/ml (*n* = 11; data not shown). In order to evaluate whether knocking out other complement components had any effect on serum C1q levels, C1q was measured in different complement KO mouse strains at 6 and 12 months of age (Table 2). As expected, *C1q* KO mouse sera gave no signal in the assay (Fig. 4A). In *C3* KO mice, serum C1q levels were significantly lower compared to WT at 6 months but were not different between the two age points (Fig. 4B, E). Similarly, in *C7* KO mice, C1q levels were significantly lower compared to WT at 6 and 12 months; however, in this mouse, strain levels were significantly lower at 12 months compared to 6 months (Fig. 4D, E). In *Crry* KO mice, C1q levels were similar to WT at 6 and 12 months and showed a small, non-significant fall at 12 months relative to 6 months (Fig. 4C, E). There were no significant differences between genders for C1q levels in any of the mouse lines (Table 2).

**Table 2:** Sample cohorts. A list of each strain and age cohort analysed in the ELISA, including the number of animals of each gender within each cohort at 3, 4, 6, 9, and 12 months (m). Mean serum and brain C1q levels are reported in microgrammes per millilitre and nanogrammes per microgramme total brain lysate protein (± sd), respectively. No significant differences in gender were found in any of the mouse lines (non-significant; ns). *n/a*, not applicable

	Genotype	Age	Male	Female	C1q (mean ± sd)	Gender difference
Serum	WT	3 m	6	4	72.45 ± 15.25	Ns
		6 m	5	5	64.76 ± 8.95	Ns
		9 m	4	6	59.91 ± 16.11	Ns
		12 m	5	7	55.54 ± 12.38	Ns
	APP^NL-G-F^	3 m	5	5	56.01 ± 16.54	Ns
		6 m	5	4	43.08 ± 6.31	Ns
		9 m	4	6	40.42 ± 12.98	Ns
		12 m	4	6	40.16 ± 9.55	Ns
	3xTg	4 m	4	0	46.95 ± 17.50	n/a
		12 m	5	0	32.86 ± 11.07	n/a
	*C3 KO*	6 m	3	4	39.41 ± 7.08	Ns
		12 m	4	3	41.92 ± 6.20	Ns
	*C7 KO*	6 m	5	4	49.51 ± 15.01	Ns
		12 m	5	5	32.84 ± 8.30	Ns
	*Crry KO*	6 m	6	6	68.31 ± 25.70	Ns
		12 m	5	4	56.87 ± 16.81	Ns
	*C1q KO*	6 m	6	5	n/a	n/a
		12 m	4	4	n/a	n/a
	Rat	3 m	11	0	97.69 ± 14.31	n/a
Brain	WT	3 m	4	4	0.285 ± 0.039	ns
		12 m	4	4	0.353 ± 0.043	ns
	APP^NL-G-F^	3 m	4	4	0.234 ± 0.013	ns
		12 m	4	4	0.386 ± 0.024	ns

### Quantification of C1q in Brain Homogenates from WT and AD Mice

**Fig. 5:**
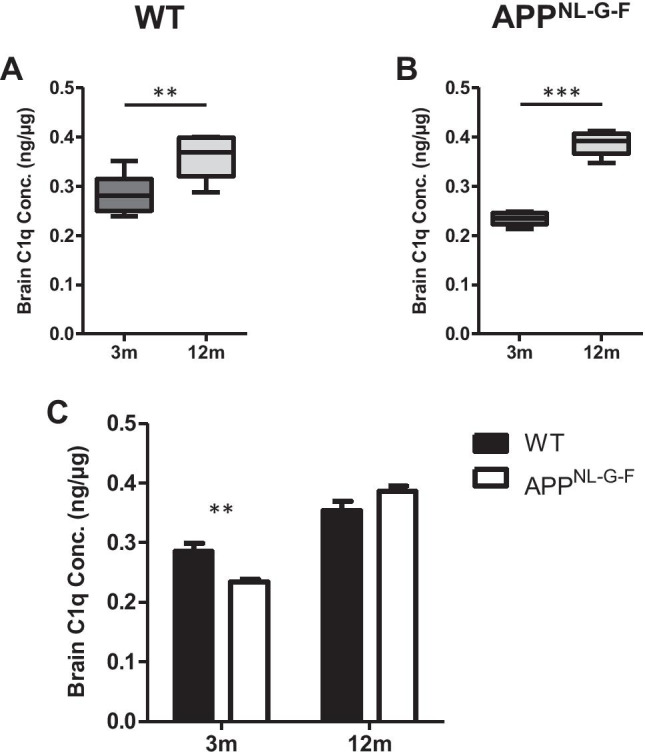
Measurement of C1q in WT and APP^NL-G-F^ mouse whole-brain lysates by quantitative sandwich ELISA. **(A)** C1q levels were measured in brain lysates from WT mice at 3 and 12 months and normalised to total protein in the lysate. Brain C1q levels were significantly elevated at 12 months compared to 3 months (*P* < 0.01). **(B)** C1q levels were measured in APP^NL-G-F^ mouse whole-brain lysates, normalised as above, at 3 and 12 months. Brain C1q levels were significantly higher at 12 months (*P* < 0.001). **(C)** Brain C1q levels were significantly lower in APP^NL-G-F^ mice than WT mice at 3 months (*P* < 0.01), but were not significantly different between the lines at 12 months. Units are nanogrammes of C1q per microgramme of total brain protein

The ELISA was used to quantify C1q levels in brain homogenates; WT and APP^NL-G-F^ mice aged 3 and 12 months were analysed (Table 2); brain C1q levels, expressed relative to total protein, were significantly elevated at 12 months compared to 3 months in both WT (Fig. 5A) and APP^NL-G-F^ mice (Fig. 5B). When strains were compared, brain C1q levels were significantly reduced in APP^NL-G-F^ mice compared to WT mice at 3 months, but not at 12 months (Fig. 5C). There were no significant differences between genders for brain C1q levels in either of the mouse lines (Table 2).

## Discussion

Although long-established as hallmarks of AD and other dementias, the mechanisms of neurodegeneration and pathological synaptic loss are yet to be fully understood. There is now a substantial body of evidence that synaptic pruning, an essential developmental process, is aberrantly reactivated in the AD brain [22, 23]. In AD, it is broadly accepted that synaptic loss is the best correlate of cognitive decline [48]. Both physiological and pathological synaptic pruning are complement-dependent processes that entail activation of the classical pathway with deposition of components C1q and C3b/iC3b onto “weak” synapses, marking them for removal by microglia [28, 29]. Precisely, what C1q binds on weak synapses, and what designates synapses as “weak”, remains unknown. Many researchers are using rodent models to investigate the role of C1q in this context; however, sensitive and reproducible methods of detecting and quantifying C1q in rodent biological fluids and pathological tissues are lacking. To address this need, we generated anti-C1q mAb in C1q-KO mice using pure mouse C1q as immunogen, and developed a quantitative, sensitive, and specific sandwich ELISA from a non-competing pair of mAb that enabled measurement of C1q levels in mouse and rat serum. The sandwich ELISA passed all the standard immunoassay tests of reproducibility and reliably quantified C1q in rodent serum and brain tissue. Serum C1q levels measured in 3-month-old WT mice aligned with previous reports [49, 50].

To test the utility of the assay in experimental models, we first examined the effect of ageing on serum C1q levels in WT and AD model mice on the C57BL/6 background. We observed a progressive and significant decrease in serum C1q with age in WT mice, significant at 12 months; APP^NL-G-F^ AD model mice also demonstrated a progressive and significant decrease in serum C1q concentrations with increasing age, more marked than in WT mice, significant at 9 and 12 months compared to 3 months. Comparison between the two strains showed decreased C1q levels in APP^NL-G-F^ mice compared to WT at each age (Fig. 3). We observed a similar decrease in plasma C1q levels in male 3xTg AD model mice that reached significance compared to WT at 12 months despite the low sample number available for analysis. These findings contradict several reports that serum C1q levels, measured using Western blotting, a semi-quantitative and insensitive method, did not change or even increased with age in C57BL/6 mice [51, 52]. One report described a substantial increase in serum C1q levels between 2 and 12 months in C57BL/6 mice via Western blotting, supported by an unspecified ELISA technique [53]. Fonseca et al., using Western blotting and densitometry, reported no significant differences in serum C1q levels at 5 and 10 months in the Arctic48 AD model compared to WT mice [52]. The fact that we used a sensitive and specific ELISA, incorporating two mAb against mouse C1q and properly validated with appropriate quality control measure, gives us confidence that the findings we report in WT and AD model mice are correct. We show that our assay accurately measures both free C1q and C1q in the C1 complex, important in contexts where the proportions of free C1q and C1 complex might vary and enabling quantification in both serum and EDTA plasma; in the latter, Ca^2+^ chelation disrupts the C1 complex.

Several studies report that serum C1q levels increase with age in healthy human donors [54, 55]. In our recent AD plasma biomarker study, there was no difference in serum C1q levels between aged healthy controls, and individuals with MCI or AD [8]. Others have reported that C1q levels are reduced in the CSF of AD patients compared to controls and suggested that this might be an AD biomarker [56]. Of note, CSF C1q levels reported in this study were ~ 0.2–0.4 μg/ml, comfortably within the working range of our assay. We were unable to source mouse CSF for the study, so to support the capacity of our assay to measure C1q in CSF, we spiked mouse C1q into human CSF at a relevant dose and showed that it could be measured in the assay with good recovery. While we are not aware of published studies measuring C1q levels in rodent CSF, the assay detects mouse C1q in an appropriate matrix (human CSF) at the low (ng/ml) levels predicted from human data to be present in rodent CSF; these data show that the described assay offers the prospect of such studies in disease models.

Next, we explored the impact of complement gene knockouts; serum C1q was measured in *C1q* KO, *C3* KO, *C7* KO, and *Crry* KO mouse models (Fig. 4). All of these lines were on the C57/BL6 background, all had been back-crossed onto the in-house C57BL/6 (WT) line, and all were maintained in the same room and under identical containment; these precautions reduce but do not eliminate risk of other genetic or environmental differences impacting the inter-line comparison. As expected, serum from C1q KO mice gave no signal in the assay, confirming the specificity of the assay and the novel mAbs reported here. Serum C1q levels were reduced in *C3* KO mice at 6 and 12 months, significantly in the former, compared with matched WT mice. Precisely, how the absence of C3 impacts C1q levels is unclear; however, collaboration between C1q and C3 is critical to modulation of innate and adaptive immunity in mice and men [57, 58]. Absence of C3 might thus favour immune dysregulation and increased C1q consumption. Crry is the dominant cell-associated C3 convertase inhibitor in rodents; deficiency of Crry causes systemic consumption of C3 and secondary C3 deficiency [36]. No differences in C1q levels were observed between WT and *Crry* KO mice at any age suggesting that secondary C3 deficiency does not have the same effect as primary C3 deficiency in the mice. In *C7* KO mice, serum C1q levels were significantly reduced at 6 and 12 months compared to matched WT mice, an unexpected finding given that absence of C7 impacts only the terminal pathway and formation of MAC. MAC plays roles in the homeostatic clearance of apoptotic cells [58, 59]; we suggest that absence of MAC may lead to an increased burden of apoptotic cells during ageing that bind C1q, well described in lupus models, leading to reduced plasma C1q levels.

Finally, in order to demonstrate whether the assay could be used to measure levels of C1q in tissue extracts, we measured the protein in mouse brain homogenate (Fig. 5). The assay performed well in brain homogenates, and we observed a significant increase in brain C1q levels at 12 months compared to 3 months in WT mice, a finding that is concordant with the literature as both C1q protein and mRNA levels have been reported to increase with age in the human and mouse brain [10, 51, 60]. We also demonstrated a significant increase in brain C1q levels in aged APP^NL-G-F^ mice, almost doubling from 3 to 12 months of age. Brain C1q levels were significantly lower in APP^NL-G-F^ mice relative to WT mice at 3 months, but because of the extent of increase with age, they were not significantly different at 12 months. Others have reported increased C1q expression in and around areas of pathology in the APP^NL-G-F^ model [61]. Our whole-brain measures would miss local changes in expression; dissection and separate analysis of key brain regions for measurement of such changes is an obvious next step. Elevated brain C1q levels at late disease stage in AD mouse models relative to matched WT mice have been reported in several other studies, mostly using Western blotting to semi-quantify C1q [28, 52, 60–62]. Although there is an abundance of immunohistochemical evidence for C1q deposition in late-stage AD brain [63, 64], we are not aware of published studies that quantitatively measure C1q protein levels in healthy and AD human brain tissue; our unpublished work suggests that the assay described here, modified to measure human C1q, could detect and quantify C1q in AD brain extracts.

Although the current work is focussed on AD models, complement-driven synaptic elimination is also reported in other neurodegenerative disorders, including MS, and in neurodevelopmental disorders such as schizophrenia. Beyond the brain, C1q is a critical factor in autoimmune and autoinflammatory diseases [65]. Hence, the availability of a novel ELISA to reliably quantify C1q levels in rodent biological fluids and tissues may have wide application in the study of models of diverse complement-driven disorders.

## Supplementary Information

ESM 1 (PPTX 1321 kb)

## Data Availability

Primary data and materials described are available on reasonable request for academic use.
