# Supplementary figures and images for: Novel Monoclonal Antibodies Against Mouse C1q: Characterisation and Development of a Quantitative ELISA for Mouse C1q
Source: Mol Neurobiol. 2021 May 18;58(9):4323–36. doi: 10.1007/s12035-021-02419-5 (PMC8487419; doi:10.1007/s12035-021-02419-5)

## Slide 1
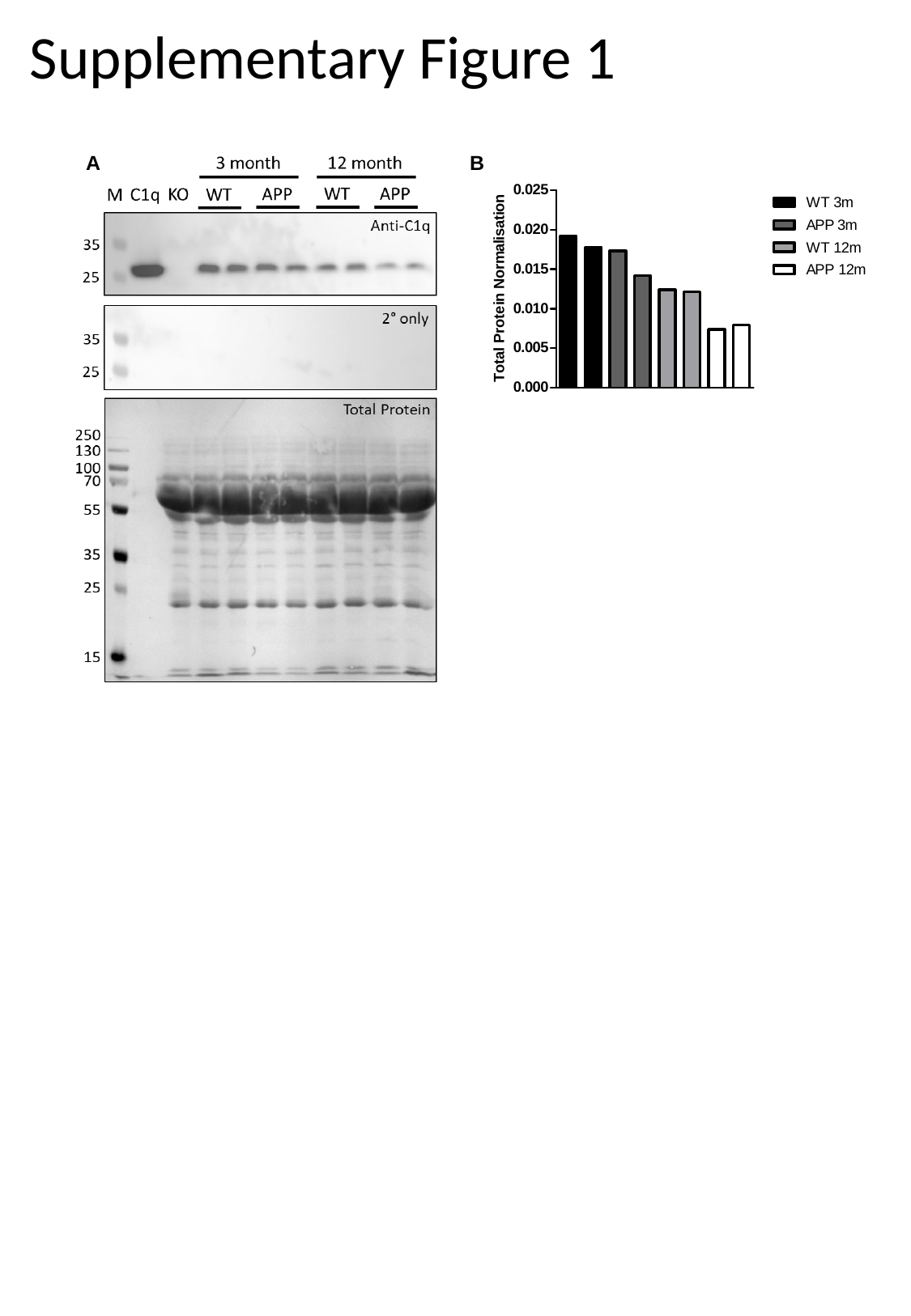

Supplementary Figure 1
A
B

## Slide 2
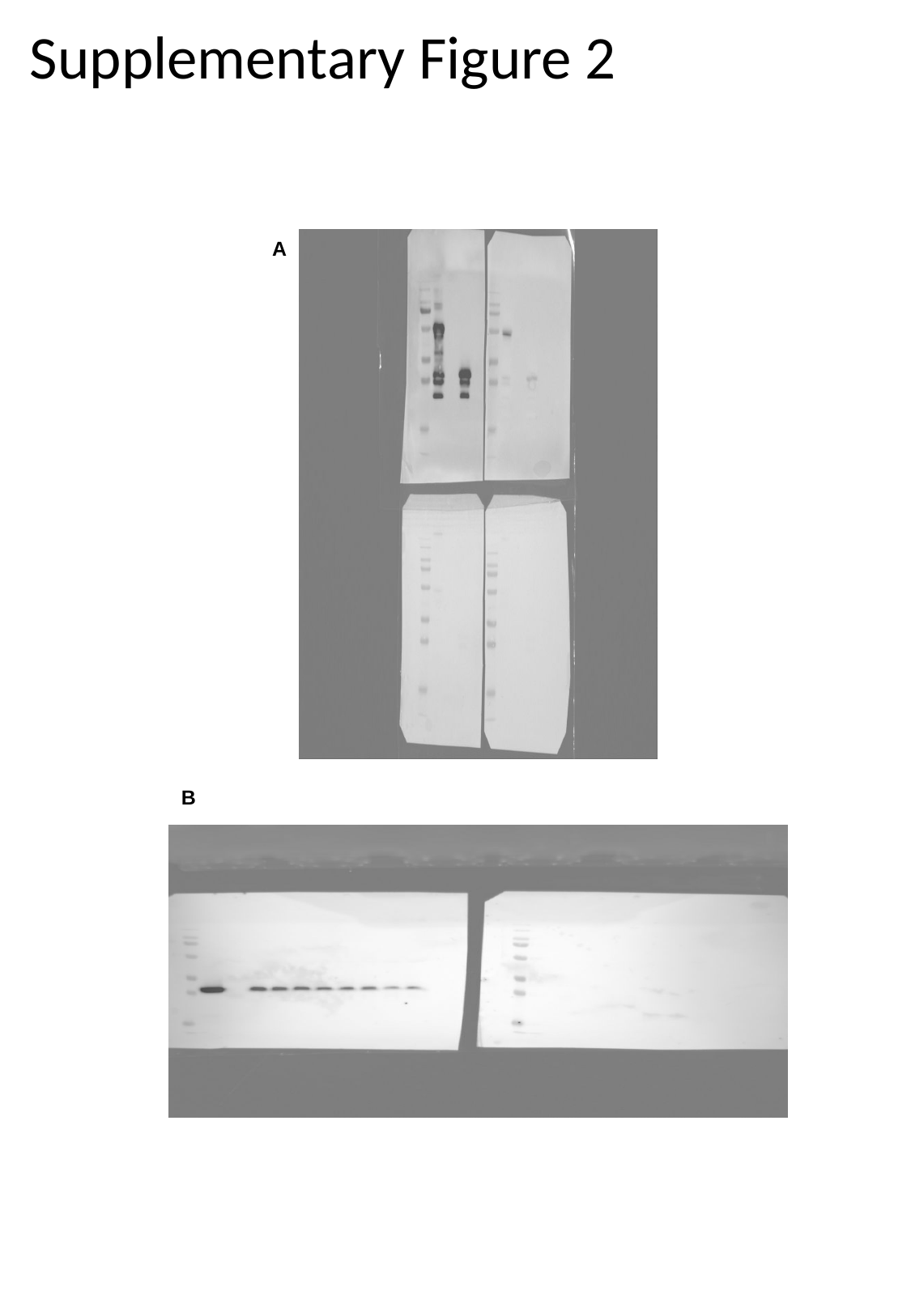

Supplementary Figure 2
A
B

Supplement: Supplementary file 1 — (PPTX 1321 kb) [file 12035_2021_2419_MOESM1_ESM.pptx]
